# Role of SHIP-1 in the Adaptive Immune Responses to Aeroallergen in the Airway

**DOI:** 10.1371/journal.pone.0014174

**Published:** 2010-11-30

**Authors:** Sukit Roongapinun, Sun-Young Oh, Fan Wu, Ampai Panthong, Tao Zheng, Zhou Zhu

**Affiliations:** 1 Division of Allergy and Clinical Immunology, The Johns Hopkins Asthma and Allergy Center, Johns Hopkins University School of Medicine, Baltimore, Maryland, United States of America; 2 Department of Pharmacology, Faculty of Medicine, Chiang Mai University, Chiang Mai, Thailand; New York University, United States of America

## Abstract

**Background:**

Th2-dominated inflammatory response in the airway is an integral component in the pathogenesis of allergic asthma. Accumulating evidence supports the notion that the phosphoinositide 3-kinase (PI3K) pathway is involved in the process. We previously reported that SHIP-1, a negative regulator of the PI3K pathway, is essential in maintaining lung immunohomeostasis, potentially through regulation of innate immune cells. However, the function of SHIP-1 in adaptive immune response in the lung has not been defined. We sought to determine the role of SHIP-1 in adaptive immunity in response to aeroallergen stimulation in the airway.

**Methodology/Principal Findings:**

SHIP-1 knockout (SHIP-1^−/−^) mice on BALB/c background were immunized with ovalbumin (OVA) plus aluminum hydroxide, a strong Th2-inducing immunization, and challenged with OVA. Airway and lung inflammation, immunoglobulin response, Th2 cytokine production and lymphocyte response were analyzed and compared with wild type mice. Even though there was mild spontaneous inflammation in the lung at baseline, SHIP-1^−/−^ mice showed altered responses, including less cell infiltration around the airways but more in the parenchyma, less mucus production, decreased Th2 cytokine production, and diminished serum OVA-specific IgE, IgG1, but not IgG2a. Naïve and OVA sensitized SHIP-1^−/−^ T cells produced a lower amount of IL-4. *In vitro* differentiated SHIP-1^−/−^ Th2 cells produced less IL-4 compared to wild type Th2 cells upon T cell receptor stimulation.

**Conclusions/Significance:**

These findings indicate that, in contrast to its role as a negative regulator in the innate immune cells, SHIP-1 acts as a positive regulator in Th2 cells in the adaptive immune response to aeroallergen. Thus any potential manipulation of SHIP-1 activity should be adjusted according to the specific immune response.

## Introduction

Asthma is a chronic inflammatory disorder of the lung with reversible airway obstruction, airway hyperresponsiveness, mucus hyperplasia, and airway remodeling [1], [2]. Th2 cytokines IL-4 and IL-13 and the STAT6 signaling pathway play a critical role in the pathogenesis of asthma. However, recent evidence has pointed to the phosphoinositide 3-kinase (PI3K) signaling as another important pathway in the generation of the asthma phenotype. PI3K and its downstream signaling molecules such as Akt are critical in a variety of biological processes, including cell proliferation, survival, and migration. PI3K is critical in T cell activation and survival [3]. The PI3K pathway is activated after allergen challenge in sensitized mice and expression of a dominant-negative PI3K subunit or use of PI3K inhibitors ameliorate the inflammatory response to allergen [4], [5], [6].

Upon activation, PI3K phosphorylates phosphatidylinositol (4,5) bisphosphate (PI(4,5)P2) to PI(3,4,5)P3, which is the main lipid second messenger for downstream signaling. The intracellular levels of PI(3,4,5)P3 are regulated by two phosphatases, tensin homologue deleted on chromosome ten (PTEN) and Src homology region 2 domain-containing inositol 5′-phosphatase-1 (SHIP-1). SHIP-1 dephosphorylates PI(3,4,5)P3 to generate PI(3,4)P2 [7], [8]. SHIP-1 is believed to be a negative regulator in a variety of cytokine, immunoreceptor, and growth factor signaling pathways in different cell types, including T cells, B cells, mast cells, basophils, and neutrophils [8], [9], [10], [11], [12], [13], [14]. SHIP-1 deficiency as in gene-targeted deletion resulted in spontaneous inflammatory cell infiltration in the lung of some mice [11], [12], which has been recently identified by our group as a Th2-like allergic inflammatory phenotype that may be related to enhanced mast cell response [15]. Adoptively transferred SHIP-1 deficient mast cells were shown to enhance allergic and anaphylactic responses *in vivo* [16]. In T cells, SHIP-1 was reported to regulate cytokine activation in a way favoring Th2 response but limiting Th1 cytotoxicity [17]. However, a regulatory role of SHIP-1 in adaptive immune response to allergen stimulation in the airway has not been established.

In this study, we examined the role of SHIP-1 in Th2 cell activation, Th2 cytokine production and allergic inflammation in the lung in response to allergen stimulation using SHIP-1 null mice in an allergic asthma model. Our results show that in the absence of SHIP-1 Th2-dominated allergic inflammatory responses to ovalbumin are impaired, suggesting a critical role of SHIP-1 in adaptive Th2 immune response to aeroallergen.

## Results

### Airway inflammatory responses to allergen in SHIP-1^−/−^ mice

**Figure 1:**
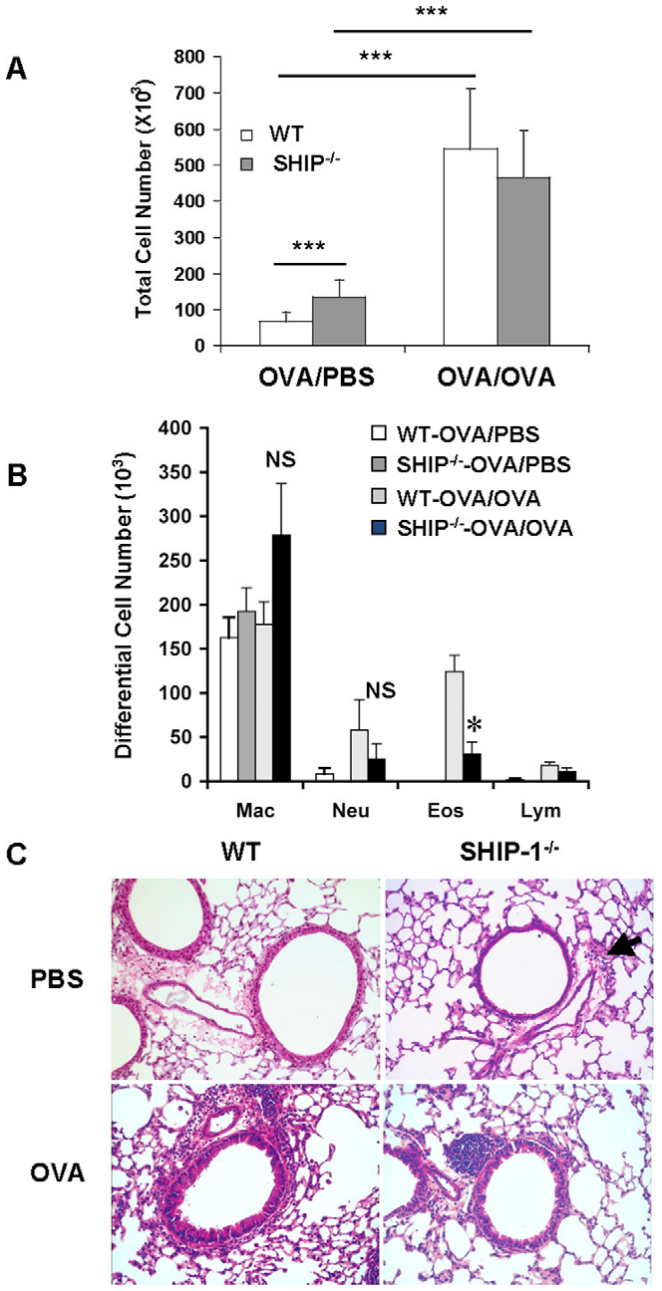
Allergic inflammatory response in the airways. WT and *SHIP-1^−/−^* mice were sensitized with OVA allergen and challenged with PBS (OVA/PBS) or OVA (OVA/OVA) as described in Methods. Total and differential cell counts in the BAL fluid were determined. (A) BAL total cell counts. (B) BAL differential cell counts. Data expressed as Mean±SEM were from a representative experiment (n = 4–6 mice each group; *p<0.05). (C) Lung histology, H&E staining (20x), with an arrow indicating inflammatory cell infiltration.

To assess lung inflammatory responses to aeroallergen we determined the total and differential cell counts in the BAL fluid 24 hr after last OVA challenge (day 16). At age of 6–8 weeks, WT mice in PBS control group had baseline total cell counts in the BAL but SHIP-1^−/−^ mice received PBS had slightly but significantly increased total cell counts in the BAL (**Figure 1A**), indicating mild spontaneous inflammation in the lung. This was consistent with our previous report on SHIP-1^−/−^ mice that develop spontaneous inflammation in the lung [15], although BALB/c SHIP-1^−/−^ mice have a relatively milder phenotype. After OVA challenge, as expected, WT mice had significantly increased BAL cell counts compared to the PBS group. SHIP-1^−/−^ mice also had increased BAL cell counts but, unexpectedly, not more than that of WT-OVA mice (**Figure 1A**). Differential cell counts showed that after OVA challenge WT mice had increased eosinophils in the airways as expected in this model. However, SHIP-1^−/−^ mice had significantly less eosinophils compared to WT-OVA mice, even though the number was higher than the baseline (**Figure 1B**). Similar trend was seen in the numbers of neutrophils and lymphocytes but the difference between WT mice and SHIP-1^−/−^ mice was not statistically significant.

### Lung histology

**Figure 2:**
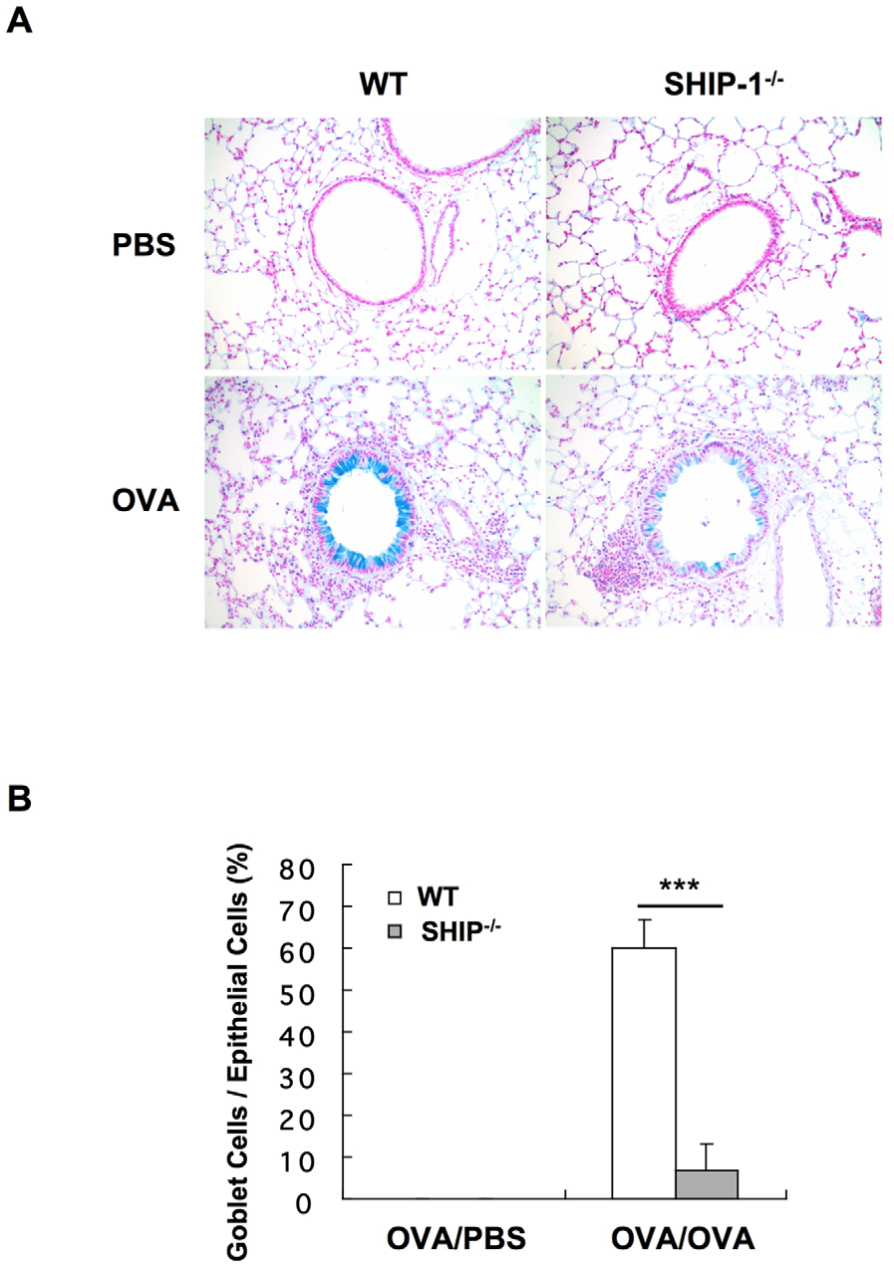
Lung histology and quantitative assessment of mucus metaplasia. (A) Alcian blue staining (20x) of lung sections. (B) Percentage of Alcian blue positive cells in the airways. Epithelial cells, 100–200/each airway, were counted and the percentage of Alcian blue positive cells was determined (n = 3–4/group).

Lung histology revealed that in PBS groups, WT mice had no inflammatory cell infiltration in the lung but SHIP-1^−/−^ mice had some cell infiltration with small clusters of cells at the vicinity of the bronchovascular bundles in the lung (**Figure 1C**). With OVA challenge, WT mice had a usual pulmonary inflammatory response with cellular infiltration surrounding the airways and vasculatures, similar to peribronchial cuffing. Most cells were eosinophils and mononuclear cells. However, with OVA challenge SHIP-1^−/−^ mice only showed a modest increase in cell infiltration in the lung and the pattern of distribution of the cells was different from that of WT mice, as most of the cells were in the lung parenchyma with some close to but not surrounding the airways (**Figure 1C**). Mucus hyperplasia is a characteristic Th2 response to allergen stimulation. We asked if SHIP-1^−/−^ mice had altered mucus response. Alcian blue stained lung sections revealed that without OVA stimulation no mucin-producing cells were found in the lung of WT or SHIP-1^−/−^ mice. After OVA challenge WT mice had a robust mucus response with a significant number of goblet cells in the airways while SHIP-1^−/−^ mice only had a minimal response (**Figure 2A**). Quantitative measurement of Alcian blue positive cells in the airways demonstrated that SHIP-1^−/−^ mice had significantly fewer mucin producing cells than WT mice in response to OVA (**Figure 2B**).

### OVA-specific IgE, IgG1 and IgG2a production

**Figure 3:**
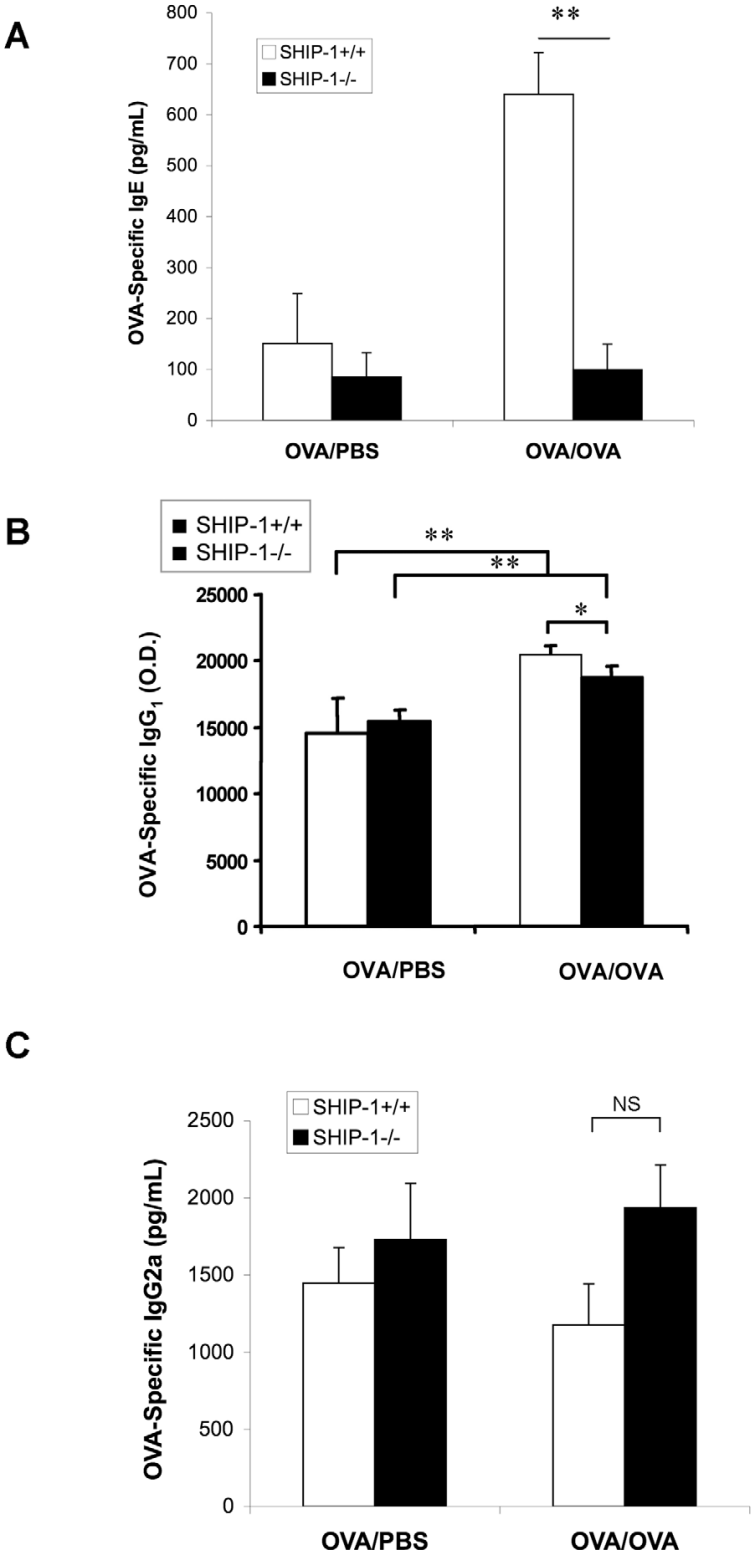
Serum levels of OVA-specific immunoglobulins. Serum samples of WT and SHIP-1^−/−^ mice were collected at the time of sacrifice and OVA allergen-specific IgE, IgG1 and IgG2a were measured by ELISA. (A) Serum OVA-specific IgE. (B) Serum OVA-specific IgG1 and (C) Serum OVA-specific IgG2a (n = 4–6 mice each group; *p<0.05 and **p<0.01).

We reported previously that SHIP-1^−/−^ mice had increased total IgE levels in the lung [15]. In this study we examined the allergen induced immunoglobulin response by measuring serum levels of OVA-specific IgE, IgG1 and IgG2a. Significant amount of antigen-specific IgE was detected in the serum samples from WT mice after OVA challenge. However, SHIP-1^−/−^ mice with OVA challenge had only background levels of OVA-specific IgE in the serum (**Figure 3A**). Even though OVA-specific IgG1 production in SHIP-1^−/−^ mice after OVA stimulation was higher than that at the baseline, the level was still significantly lower than that of WT mice (**Figure 3B**). These results indicate an impaired antibody specific response to allergen in the SHIP-1^−/−^ mice. After OVA challenge neither WT mice nor SHIP-1^−/−^ mice showed any change in the serum levels of IgG2a, which is usually involved in Th1 responses (**Figure 3C**).

### Decreased Th2 cytokine production in the lung

**Figure 4:**
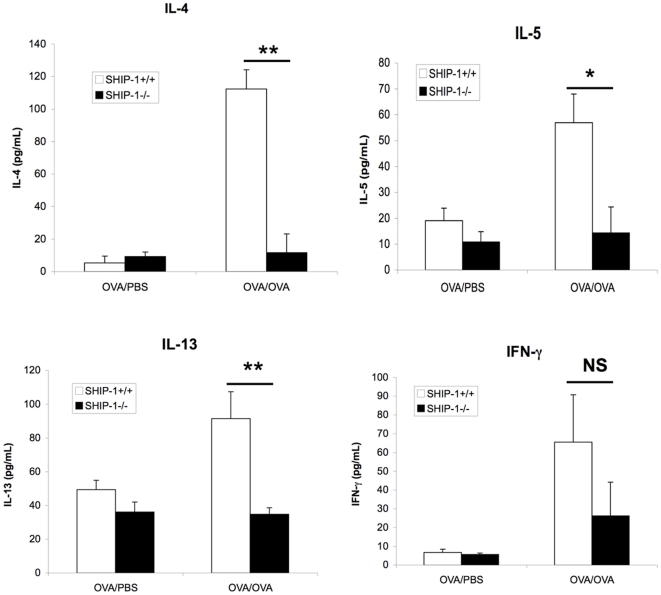
Inflammatory cytokines in the BAL fluid. After sensitization with OVA and challenged with OVA or PBS control, BAL fluid was obtained and cytokines in the BAL were measured using ELISA kits. Data were Mean±SEM (NS, not significant; *p<0.05; **p<0.01. n = 4–6 mice each group).

Next we examined the cytokine response to OVA allergen challenge in the airway. WT mice and SHIP-1^−/−^ mice treated with PBS showed only background levels of Th2 cytokines IL-4, IL-5, and IL-13 and Th1 cytokine IFN-γ in the BAL. WT mice challenged with OVA showed significant increases in Th2 cytokines. In contrast, SHIP-1^−/−^ mice challenged with OVA did not show any increase in IL-4, IL-5, or IL-13 in the BAL above the baseline. Although OVA challenged SHIP-1^−/−^ mice had a significant increase in the IFN-γ levels compared to the PBS control group and less increased IFN-γ in SHIP-1^−/−^-OVA group, the difference between WT and SHIP-1^−/−^ OVA groups was not significant (**Figure 4**).

### Reduced IL-4 production by sensitized SHIP-1^−/−^ splenic cells

**Figure 5:**
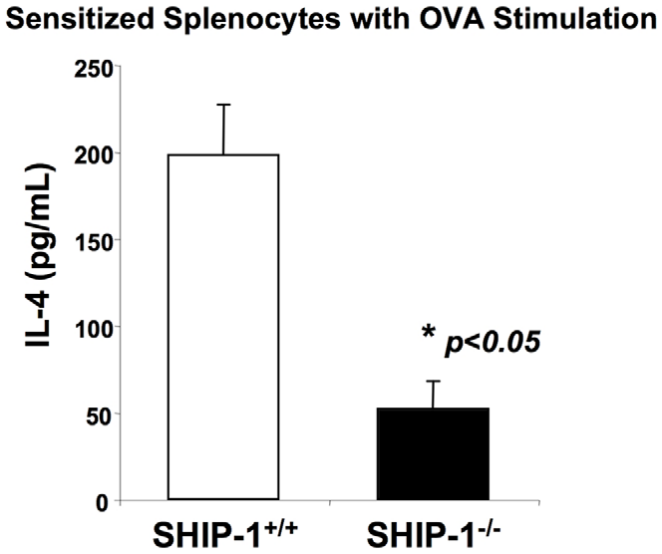
IL-4 production by sensitized splenocytes. Splenocytes were isolated from OVA sensitized and challenged WT and SHIP-1^−/−^ mice. After OVA allergen stimulation, IL-4 levels in the culture supernatant were determined (n = 6 mice for each group).

We next examined whether *in vivo* sensitized SHIP-1^−/−^ lymphocytes had a different pattern of IL-4 production relative to that of WT mice in response to OVA stimulation. Splenocytes in single-cell suspension from OVA sensitized and challenged mice were stimulated with OVA for 3 days and IL-4 in the culture supernatant was assayed. WT splenocytes responded to OVA normally and produced a significant amount of IL-4. In contrast, SHIP-1^−/−^ splenocytes produced markedly lower levels of IL-4 (**Figure 5**). This result is consistent with the observation of diminished Th2 cytokine production in the lung and suggests a defect in Th2 cell differentiation and/or Th2 cytokine production in SHIP-1^−/−^ mice.

### Decreased IL-4 production by naïve T cells from SHIP-1^−/−^ mice

**Figure 6:**
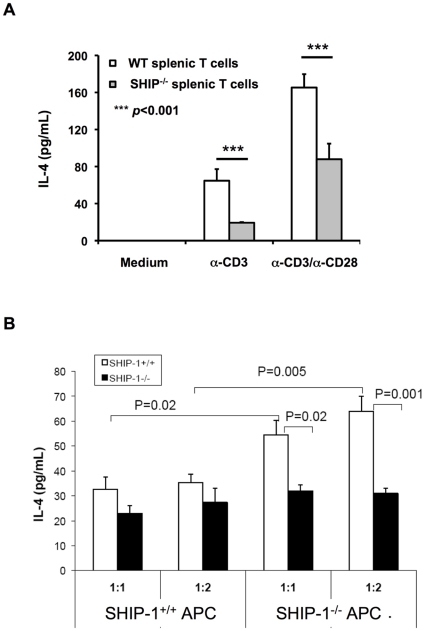
Response of naïve T cells to stimulation. (A) CD4+ T cells isolated from unsensitized WT and SHIP-1^−/−^ mice were incubated with medium, α-CD3, or α-CD3/α-CD28 for 24 hr and the levels of IL-4 in the culture medium were determined by ELISA (n = 5 each group, ***p<0.001). (B) Naïve CD4+ T cells isolated from WT and SHIP-1^−/−^ mice were incubated with negatively selected, irradiated APC from the spleen of WT or SHIP-1^−/−^ mice in different ratios in the presence of OVA_323–339_ and IL-4 production was determined (n = 4 for each group).

To further identify the cell types that were defective in response to allergen stimulation we tested T cells and APC in separate experiments. Splenic T cells from unsensitized WT and SHIP-1^−/−^ mice were prepared and activation of TCR via plate-coated α-CD3 with or without soluble α-CD28 for 72 hr was performed. SHIP-1^−/−^ T cells produced significantly lower amount of IL-4 compared with WT T cells (**Figure 6A**). These results suggest that the number of T cells able to produce IL-4 and/or the TCR signaling for IL-4 production in SHIP-1^−/−^ mice was decreased as compared to that of WT mice. Isolated T cells from WT and SHIP-1^−/−^ mice were then incubated with irradiated splenic APC from either WT or SHIP-1^−/−^ mice in the presence of OVA peptide_323–339_. Again, T cells from SHIP-1^−/−^ mice did not respond well to either WT APC or SHIP-1^−/−^ APC. Interestingly, WT T cells produced more IL-4 when incubated with APC from SHIP-1^−/−^ mice (**Figure 6B**). These results indicate that SHIP-1^−/−^ T cells had impaired response to antigen stimulation while SHIP-1^−/−^ APC had enhanced activity in the presence of antigen.

### *In vitro* Th2 differentiation and cytokine production

**Figure 7:**
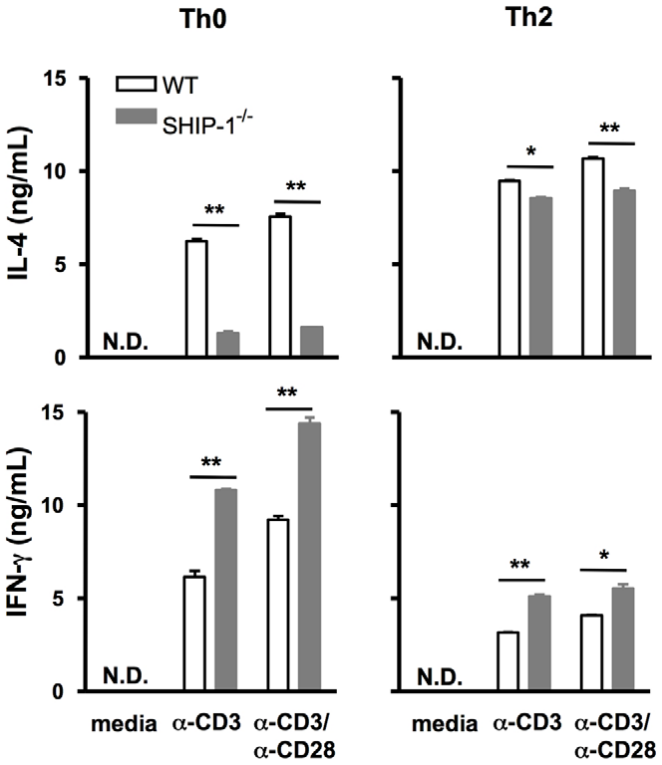
Cytokine response of Th0 and *in vitro* differentiated Th2 cells. CD4+ T cells were isolated from spleen of WT and SHIP-1^−/−^ mice and cultured (1×10^6^/well) under Th0 or Th2 polarizing conditions for 3 days. After further expanding in IL-2 for another 3 days, the cells were re-stimulated with α-CD3 or α-CD3/α-CD28 for 24 hr. IFN-γ and IL-4 levels in the culture supernatant were determined by ELISA. The results are shown as Mean±SEM of triplicate samples of pooled cells from 2 mice per group. The same experiment was repeated two more times with similar results (N.D. = not detected; *p<0.05; **p<0.01).

The above results indicate that T cells from SHIP-1^−/−^ mice undergoing Th2 differentiation *in vivo* had suppressed IL-4 production. To verify whether this is differentiation related we performed *in vitro* Th2 differentiation experiments. Splenic T cells from naïve mice were incubated under the Th2-polarizing condition and T cell production of IL-4 and IFN-γ was determined after the cells were re-stimulated with medium control, α-CD3 or α-CD3/α-CD28. Without TCR stimulation undifferentiated Th0 cells and differentiated Th2 cells from WT and SHIP-1^−/−^ mice did not produce any detectable levels of IL-4 or IFN-γ (**Figure 7**). With TCR stimulation WT Th0 cells produced a considerable amount of IL-4 that was further increased by differentiated Th2 cells. In contrast, both SHIP-1^−/−^ Th0 and Th2 cells produced significantly lower amounts of IL-4 compared to those of WT cells (**Figure 7**). On the other hand, the opposite was observed in IFN-γ production in that SHIP-1^−/−^ Th0 and Th2 cells produced increased IFN-γ than those of WT cells (**Figure 7**). These studies indicate that SHIP-1^−/−^ Th0 cells can differentiate into Th2 cells but to a suboptimal degree and SHIP-1 is critical for T cell IL-4 production and is a negative regulator in IFN-γ production.

## Discussion

Several studies showed that the PI3 kinase signaling pathway, particularly the PI3Kδ and PI3Kγ isoforms, plays an important role in the pathogenesis of allergen induced inflammatory response [4], [5], [6], [18], [19]. SHIP-1 regulates PI3 kinase signaling by dephosphorylating intracellular PIP3. SHIP-1 functions in a variety of cell types, including T cells and mast cells. SHIP-1 knockout mice at age 9-11 weeks developed a spontaneous Th2-like allergic inflammatory response in the lung that may involve innate immune cells [15]. In contrast, conditional deletion of SHIP-1 in T cells resulted in reduced Th2 responses to nitrophenylacetyl hapten conjugated to chicken γ globulin (NP-CGG) or to *Schistosoma* eggs [17]. The reason for these seemingly contradictory results is unknown. A possible explanation is that SHIP-1 has divergent roles in different innate and adaptive immune cells. A potential role of SHIP-1 in Th2 adaptive immune responses to aeroallergen stimulation has not been defined. In this study, we examined the role of SHIP-1 in adaptive immune response using SHIP-1 null mice in an allergic asthma model.

Upon OVA allergen challenge, sensitized WT mice displayed robust allergic inflammatory responses, including macrophage and eosinophil dominated cell infiltration in the airway and in the lung parenchyma, goblet cell hyperplasia, OVA-specific IgE and IgG1 production, and Th2 cytokine production in the lung. In contrast, in response to OVA challenge, sensitized SHIP-1^−/−^ mice showed similar or lower cell counts in the BAL, less infiltration in the lung tissue, less mucus production, no OVA-specific IgE and lower IgG1 in the serum, and no increased production of Th2 cytokines, IL-4, IL-5, and IL-13 in the lung. These results were surprising in that SHIP-1^−/−^ mice on C57BL/6x129sv mixed background developed spontaneous Th2-like inflammation in the lung as described in our previous study [15] and SHIP-1^−/−^ mice on BALB/c background at age 6–8 weeks showed mild but detectable cell infiltration in the airway in the present study. However, these two studies were set to answer different questions about the functions of SHIP-1. One is concerning spontaneous Th2-like inflammation without exposure to known allergens with the possibility of involving mast cells or basophils that are able to produce Th2 cytokines. Another is to examine the role of SHIP-1 in the adaptive immune response in an allergic inflammation model induced by OVA allergen. The latter was carried out in younger mice on BALB/c background, which are usually more prone to Th2 immunity, to test T cell and B cell responses.

Indeed, our experiments testing T cell responses showed that SHIP-1^−/−^ splenocytes isolated from OVA sensitized and challenged mice were much less responsive to OVA antigen stimulation in IL-4 production compared to WT cells. Furthermore, isolated naive SHIP-1^−/−^ T cells were less responsive to TCR stimulation, although SHIP-1^−/−^ APC might have increased activity. These results suggest that either the development of Th2 cell population in SHIP-1^−/−^ mice is retarded or the IL-4 producing capacity of SHIP-1^−/−^ T cells is decreased. The mechanism for enhanced APC activity in SHIP-1 deficient cells is under investigation.

Further *in vitro* differentiation studies demonstrated that compared to WT cells, undifferentiated SHIP-1^−/−^ Th0 cells produced significantly less IL-4 and these cells could be polarized to Th2 cells but to a limited degree. These findings indicate that SHIP-1 is essential for optimal Th2 development and response to antigen stimulation. Interestingly, IFN-γ production in SHIP-1^−/−^ T cells was significantly increased under both Th0 and Th2 conditions (Figure 7). This is consistent with the findings reported by Tarasenko et al. suggesting that SHIP-1 may have inhibitory effect on IFN-γ production [17]. On the other hand, this data seems to be different from our in vivo findings that the level of BAL IFN-γ of SHIP-1^−/−^ mice had a trend of reduction compared to WT mice. Although IFN-γ is not a reliable cytokine in OVA induced Th2 responses, a possible explanation is that the in vivo finding is a result of impaired T cell response, involving both IFN-γ and Th2 cytokines.

The finding that the level of OVA-specific IgE in SHIP-1^−/−^ mice was diminished seems in contrast with our previous observation of increased total serum IgE in the SHIP-1^−/−^ mice on the mixed background. The reason for the difference is not known. In addition, the reduced OVA-specific IgE production may have some parallels in a previous study in which inhibition of p110δ PI3 kinase genetically or pharmacologically enhanced IgE production after OVA allergen immunization [20]. This is paradoxical with the observation that OVA induced Th2 cytokine production and allergic inflammatory response was significantly reduced in p110δ null mice or in the presence of a p110δ inhibitor [5], [18]. The former study and ours indicate that the intracellular levels of PIP3 are important in regulating IgE production in response to allergen stimulation.

Our findings of an incomplete Th2 response to aeroallergen stimulation in SHIP-1^−/−^ mice are consistent with those in an earlier report using a different approach in different models [17]. In that study, Tarasenko et al. reported that SHIP-1 deficient T cells did not skew efficiently to Th2 *in vitro* and mice with these SHIP-1 deficient T cells showed either poor antibody response to NP-CGG immunization or diminished Th2 cytokine production in response to *Schistosoma* parasite challenge [17]. Together, this study and ours indicate that SHIP-1 is critical in Th2 development in adaptive immune response to different stimuli.

In a recent study, adoptive transfer of bone marrow-derived mast cells from SHIP-1^−/−^ mice enhanced OVA allergen induced inflammatory response in mast cell deficient recipients [16]. In that system, T cells, B cells, and APC in the adaptive immune system still have intact SHIP-1 function and can mount an efficient Th2 immune response. Thus in this case, SHIP-1 deficient mast cells contribute to the process as effector cells that amplify the Th2 response. This is in accordance with our previous study [15] and is not contradictory to the findings in the present study or to those on SHIP-1 deficient T cells [17].

Taken together, our studies on spontaneous inflammation in the lung and on adaptive immune responses to allergen stimulation in SHIP-1 deficient mice demonstrate that SHIP-1 plays divergent roles in mast cells and T cells in innate and adaptive immune responses, respectively, under different circumstances. Thus caution should be taken in the development of regimens targeting SHIP-1. Non-specific promotion or suppression of SHIP-1 activity may not achieve the desired outcome of treating Th2 dominated diseases such as allergy, anaphylaxis and asthma. Instead, cell-specific approach at different stages should be considered.

## Materials and Methods

### Animals

The SHIP-1^−/−^ mice were generated as previously described [10]. We reported in a previous study that SHIP-1^−/−^ mice on a C57BL/6x129Sv mixed genetic background developed spontaneous and progressive Th2-like allergic inflammatory responses in the lung [15]. SHIP-1^−/−^ mice on BALB/c genetic background develop similar but milder inflammatory responses in the lung with slower disease progression. In this study 6–8 week old SHIP-1^−/−^ mice on BALB/c genetic background (backcrossed more than 10 generations from the original knockout mice) were used. In all experiments, SHIP-1^−/−^ mice were compared with age-matched wild type (WT or SHIP-1^+/+^) mice. Mice were maintained in a specific pathogen-free facility. All experimental protocols involving mice were approved by the Johns Hopkins University Institutional Animal Use and Care Committee.

### OVA allergen immunization and challenge

Mice were immunized by intraperitoneal (i.p.) injection of 75 µg ovalbumin (OVA) mixed with 1 mg aluminum hydroxide (Alum) (Sigma-Aldrich, St. Louis, MO) in a volume of 200 µL on days 0 and 7 and challenged by intranasal (i.n.) instillation of 20 µg OVA in 20 µl PBS (OVA/OVA group) or PBS alone (OVA/PBS group) under anesthesia on days 14 and 15. Prior to application, OVA preparation was depleted of endotoxin by passing through Detoxi-Gel columns (Pierce, Rockford, IL). On day 16 mice were sacrificed and bronchoalveolar lavage (BAL) fluid was collected in 3×0.7 ml PBS as previously described [21]. Total cell counts in the BAL fluid were determined using a Neubauer hemocytometer. BAL cell differentials (200 counts/slide) were determined after centrifugation of 1×10^5^ cells in Cytospin II (Thermo Shandon, Pittsburgh, PA). Supernatants from the BAL samples were kept at −70°C for later cytokine measurement.

### Lung histology and quantitative assessment of mucus hyperplasia

After obtaining the BAL samples, the lungs were perfused and then removed en bloc and inflated with and fixed in 10% buffered formalin (Sigma-Aldrich) overnight before being processed and embedded in paraffin as described previously [21]. Embedded lungs were sectioned at 5 µm thickness and stained with H&E and Alcian blue for evaluation of lung tissue inflammation and airway mucous metaplasia, respectively [22]. Quantitative assessment of mucus hyperplasia was performed by counting Alcian blue positive cells in the epithelia of 5–7 middle-sized airways of each sample. The percentage of Alcian blue positive cells/total epithelial cells was calculated.

### Preparation of CD4+ T cells, antigen presenting cells (APC), and stimulation of T cells

Splenic CD4+ T cells were isolated through negative selection using the CD4 T lymphocyte enrichment IMag set (BD Pharmingen, San Diego, CA). The purity of the T cells was greater than 92% by flow cytometry analysis. To isolate APC, splenocytes were depleted of CD4+ and CD8+ cells using an IMag set (BD Pharmingen). T cell-depleted APCs were then irradiated with 3500 rad before the co-culture experiments. OVA peptide 323–339 (OVA_323–339_) (5 µg/ml) was added to the T cell culture in the presence of APCs [23]. The ratio of T cell to APC was 1∶1 or 1∶2. In other experiments, purified T cells (1×10^5^/well) were incubated for 3 days with plate-bound anti-CD3 (10 µg/ml, BD Pharmingen) and soluble anti-CD28 (2 µg/ml, eBioscience, San Diego, CA). Cytokines in the culture supernatant were measured by ELISA.

To induce Th2 differentiation, T cells were isolated from spleen and incubated (1×10^6^/well) with Th2 differentiation cocktail: IL-4 (15 ng/ml, R&D Systems, Minneapolis, MN), anti-IL-12 mAb (15 µg/ml, clone C17.8, BD Pharmingen), and anti-IFN-γ mAb (15 µg/mL, clone XMG1.2, BD Pharmingen). After 72 hr the cells were washed 3 times, cultured in medium containing IL-2 (10 ng/ml) for another 3 days. After washing 3 times, the cells were re-stimulated with α-CD3, α-CD3/α-CD28, or medium control for 24 hr and cytokines in the supernatant were measured by ELISA.

### OVA-specific IgE, IgG1 and IgG2a

Serum levels of OVA-specific IgE, IgG1 and IgG2a were determined by ELISA. Briefly, plates were pre-coated overnight at 4°C with 20 µg/ml OVA in PBS. After the plates were blocked with 20% FBS, serum samples were added and incubated for 2 hr at room temperature followed by biotinylated anti-mouse IgE, IgG1 or IgG2a (BD Pharmingen), avidin-HRP, and tetramethylbenzidine substrate. The optical density of the reactions was measured by a microplate reader at 450 nm. Purified mouse IgE or IgG2a was used as standards (BD Pharmingen). IgG1 levels were expressed in O.D.

### Cytokine measurement

Cytokines IL-4, IL-5, IL-13, and IFN-γ in the BAL or in culture supernatant samples were determined by using commercially available ELISA kits according to the manufacturer's instructions (R&D Systems or eBioscience).

### Statistical analysis

Data from the experiments were analyzed by Student's t test for comparison between two groups or one-way analysis of variance (ANOVA) for comparison of more than two groups. The results were expressed as Mean±Standard Error of the Mean (SEM). Differences between groups with P values less than 0.05 were considered statistically significant.

## References

[1] Cutz E, Levison H, Cooper DM (1978). Ultrastructure of airways in children with asthma.. Histopathology.

[2] Bochner BS, Undem BJ, Lichtenstein LM (1994). Immunological aspects of allergic asthma.. Annu Rev Immunol.

[3] Okkenhaug K, Bilancio A, Emery JL, Vanhaesebroeck B (2004). Phosphoinositide 3-kinase in T cell activation and survival.. Biochem Soc Trans.

[4] Myou S, Leff AR, Myo S, Boetticher E, Tong J (2003). Blockade of inflammation and airway hyperresponsiveness in immune-sensitized mice by dominant-negative phosphoinositide 3-kinase-TAT.. J Exp Med.

[5] Lee KS, Lee HK, Hayflick JS, Lee YC, Puri KD (2006). Inhibition of phosphoinositide 3-kinase delta attenuates allergic airway inflammation and hyperresponsiveness in murine asthma model.. FASEB J.

[6] Duan W, Aguinaldo Datiles AM, Leung BP, Vlahos CJ, Wong WS (2005). An anti-inflammatory role for a phosphoinositide 3-kinase inhibitor LY294002 in a mouse asthma model.. Int Immunopharmacol.

[7] Rohrschneider LR, Fuller JF, Wolf I, Liu Y, Lucas DM (2000). Structure, function, and biology of SHIP proteins.. Genes Dev.

[8] Freeburn RW, Wright KL, Burgess SJ, Astoul E, Cantrell DA (2002). Evidence that SHIP-1 contributes to phosphatidylinositol 3,4,5-trisphosphate metabolism in T lymphocytes and can regulate novel phosphoinositide 3-kinase effectors.. J Immunol.

[9] Ono M, Bolland S, Tempst P, Ravetch JV (1996). Role of the inositol phosphatase SHIP in negative regulation of the immune system by the receptor Fc(gamma)RIIB.. Nature.

[10] Liu Q, Oliveira-Dos-Santos AJ, Mariathasan S, Bouchard D, Jones J (1998). The inositol polyphosphate 5-phosphatase ship is a crucial negative regulator of B cell antigen receptor signaling.. J Exp Med.

[11] Helgason CD, Damen JE, Rosten P, Grewal R, Sorensen P (1998). Targeted disruption of SHIP leads to hemopoietic perturbations, lung pathology, and a shortened life span.. Genes Dev.

[12] Liu Q, Sasaki T, Kozieradzki I, Wakeham A, Itie A (1999). SHIP is a negative regulator of growth factor receptor-mediated PKB/Akt activation and myeloid cell survival.. Genes Dev.

[13] MacDonald S, Vonakis B (2002). Association of the Src homology 2 domain-containing inositol 5′ phosphatase (SHIP) to releasability in human basophils.. Mol Immunol.

[14] Daigle I, Yousefi S, Colonna M, Green DR, Simon HU (2002). Death receptors bind SHP-1 and block cytokine-induced anti-apoptotic signaling in neutrophils.. Nat Med.

[15] Oh SY, Zheng T, Bailey ML, Barber DL, Schroeder JT (2007). Src homology 2 domain-containing inositol 5-phosphatase 1 deficiency leads to a spontaneous allergic inflammation in the murine lung.. J Allergy Clin Immunol.

[16] Haddon DJ, Antignano F, Hughes MR, Blanchet MR, Zbytnuik L (2009). SHIP1 is a repressor of mast cell hyperplasia, cytokine production, and allergic inflammation in vivo.. J Immunol.

[17] Tarasenko T, Kole HK, Chi AW, Mentink-Kane MM, Wynn TA (2007). T cell-specific deletion of the inositol phosphatase SHIP reveals its role in regulating Th1/Th2 and cytotoxic responses.. Proc Natl Acad Sci U S A.

[18] Nashed BF, Zhang T, Al-Alwan M, Srinivasan G, Halayko AJ (2007). Role of the phosphoinositide 3-kinase p110delta in generation of type 2 cytokine responses and allergic airway inflammation.. Eur J Immunol.

[19] Pinho V, Souza DG, Barsante MM, Hamer FP, De Freitas MS (2005). Phosphoinositide-3 kinases critically regulate the recruitment and survival of eosinophils in vivo: importance for the resolution of allergic inflammation.. J Leukoc Biol.

[20] Zhang TT, Okkenhaug K, Nashed BF, Puri KD, Knight ZA (2008). Genetic or pharmaceutical blockade of p110delta phosphoinositide 3-kinase enhances IgE production.. J Allergy Clin Immunol.

[21] Zhu Z, Homer RJ, Wang Z, Chen Q, Geba GP (1999). Pulmonary expression of interleukin-13 causes inflammation, mucus hypersecretion, subepithelial fibrosis, physiologic abnormalities, and eotaxin production.. J Clin Invest.

[22] Zhu Z, Zheng T, Homer RJ, Kim YK, Chen NY (2004). Acidic mammalian chitinase in asthmatic Th2 inflammation and IL-13 pathway activation.. Science.

[23] Cohn L, Homer RJ, Niu N, Bottomly K (1999). T helper 1 cells and interferon gamma regulate allergic airway inflammation and mucus production.. J Exp Med.

